# *SMARCA4*-related Coffin-Siris syndrome in newborn: a case report and literature review

**DOI:** 10.3389/fped.2024.1493380

**Published:** 2025-01-21

**Authors:** Yuqian Wang, Li Zhang, Jing Zhu, Liu Yang, Chan Wang, Ning Zou

**Affiliations:** Department of Pediatrics, The Second Hospital of Dalian Medical University, Dalian, Liaoning, China

**Keywords:** Coffin-Siris syndrome, *SMARCA4*, genetic abnormality, whole-exome sequencing, otorhinolaryngologic malformations

## Abstract

**Objective:**

Our objective was to examine the clinical and genetic features of Coffin-Siris syndrome resulting from a pathogenic variant in the *SMARCA4* gene.

**Methods:**

The clinical data and molecular genetic test results of a newbron with Coffin-Siris syndrome involving a pathogenic variant in the *SMARCA4* gene were retrospectively analyzed, and the related literatures were reviewed.

**Results:**

A newborn exhibited inspiratory dyspnea following birth and developmental anomalies (coarse appearance, thick hair, long eyelashes, broad nasal tip, flat nasal bridge, thin upper lip, thick lower lip, digital anomalies, cleft palate, supraglottic laryngeal chondromalacia, stenosis of the left upper bronchus and hypotonia). Whole exome sequencing revealed a heterozygous missense variant in *SMARCA4* gene (NM_003072.5 c.3127C > T, p.Arg1043Trp). Parents did not find the above pathogenic variant, which was a new pathogenic variant. In addition to our case, we also retrieved 22 cases of Coffin-Siris Syndrome in *SMARCA4* gene variation, which is a congenital multi-system dysfunction syndrome characterized by abnormal appearance and developmental retardation. The common otolaryngologic features of 23 patients with CSS in *SMARCA4* gene variant included palate abnormalities, feeding difficulties, ear abnormalities and hearing loss.

**Conclusion:**

Coffin-Siris syndrome is a rare genetic disease inherited in an autosomal-dominated manner. It is often associated with malformations in the otorhinolaryngologic system. This case has many common features with previously reported CSS cases with pathogenic variant in the *SMARCA4* gene, which further characterizes the performance of the pathogenic variant, suggesting that palatal abnormalities may be a significant feature of the genotype. For patients with developmental abnormalities, whole-genome sequencing or whole-exome sequencing is particularly important to assist diagnosis. Currently, there is no known treatment for CSS, and individuals with CSS experience various complications affecting multiple systems.

## 1 Introduction

Coffin-Siris syndrome (CSS) is a rare genetic disorder that follows an autosomal dominant inheritance pattern (1–3). This condition arises from harmful pathogenic variants in genes that encode the BAF (BRG1-related factor) chromatin mimic complex along with its associated transcription factor proteins (2). Among the 12 genes responsible for CSS, *ARID1B* is by far the most prevalent (about 60% of molecularly diagnosed cases), *SMARCB1* and *SMARCA4* are more common (∼10%) followed by *ARID1A* (∼7%) (4–6). Other genes may be responsible for the non-classical form of CSS. The global prevalence rate is about 1:10,000–1:100,000 (7). Coffin-siris syndrome is a congenital multi-system dysfunction syndrome characterized by abnormal appearance and developmental retardation. The clinical phenotypes of CSS are diverse. The typical clinical manifestations are growth retardation, special facial features (rough face, flat nose, hairline, thick eyebrows, long eyelashes, cleft lip, thick lips), hairy, hypotonia, fifth finger/toe dysplasia, etc., which can be combined with corpus callosum dysplasia (8). There exists a propensity for misdiagnosis with respect to various genetic metabolic disorders, including linear granulosis and lysosomal storage disease, among others. Additional associated characteristics include stunting, which encompasses language, movement, and cognitive development, as well as challenges with eating and a deceleration in growth. Additionally, there may be abnormalities in the heart, gastrointestinal tract, genitourinary system, ear, throat, and central nervous system (CNS) (9, 10). CSS patients present with several diseases treated by otorhinolaryngologists, including palate abnormalities, laryngotracheomalacia, and hearing loss. Through the whole exome sequencing technique, we identified one newborn diagnosed with CSS by *SMARCA4* variant. She had otolaryngologic malformations. We reported this case.

## 2 Clinical report

### 2.1 Case report

**Figure 1:**
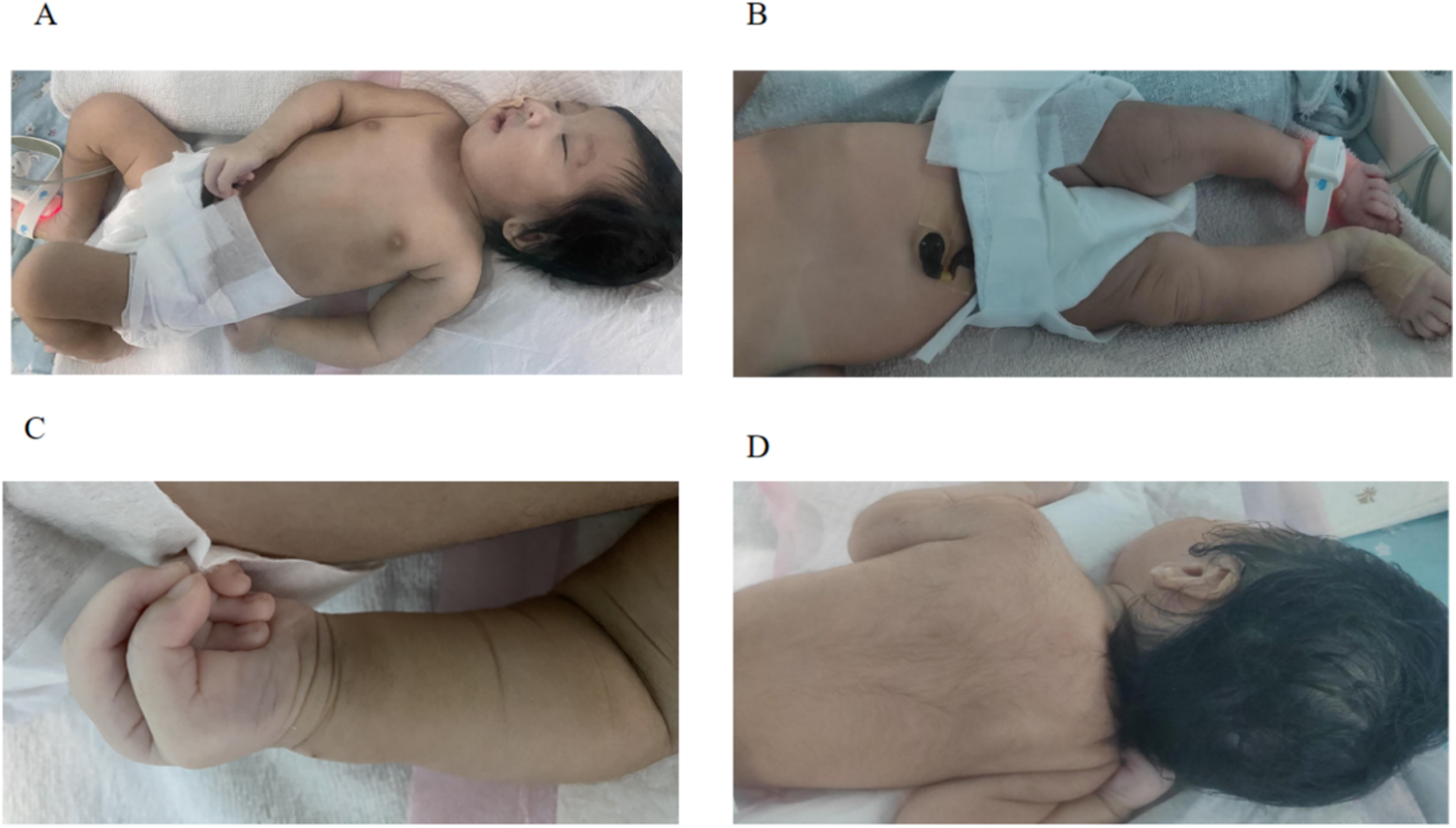
**(A–D)** The characteristics of the newborn's abnormal appearance.

An infant having trouble breathing immediately after birth was admitted to the Neonatal Intensive Care Unit (NICU) for medical intervention. She persisted in experiencing a raspy voice following childbirth. Upon admission, the physical examination revealed digital anomalies, coarse appearance, dense hair, long eyelashes, broad nasal tip, flat nasal bridge, thin upper lip, thick lip, bipedal valgus, and hypotonia (Figures 1A–D). The digital anomalies were characterized by a significant increase in the length of the distal phalanx of the thumb, and the overall length of the thumb is close to that of the other four fingers. The postnatal physical examination revealed an incomplete soft palate with an inverted “V” shape and the uvula was absent.

The mother was pregnant for the third time, but the infant was her first baby. The patient was a gestational age of 40 weeks girl, weighing 3.03 kg (10–25th percentile). The prenatal process is straightforward. The prenatal ultrasound revealed an unclear fetal septum pellucidum, yet the delivery proceeded successfully. She was born with 1 degree contamination of amniotic fluid. The Apgar score was 7 at 1 min, 7 at 5 min, and 8 at 10 min postpartum. The umbilical blood gas was within normal parameters. There is no noteworthy or remarkable family history.

Some blood test results of this patient were as follows. Blood gas: PH 7.50, PCO2 47.20 mmHg, PO2 60 mmHg, ABE 12.50 mmol/L, SBE 12.70 mmol/L, HCO3- 36.80 mmol/L, Lac 1.70 mmol/L; Blood ammonia:15 umol/L; Aldosterone: 424.43 pg/ml; Cortisol: 97.27 nmol/L.

During NICU hospitalization, the child had obvious inspiratory dyspnea. Considering upper respiratory tract obstruction, ventilator-assisted ventilation under tracheal intubation is required. Removing tracheal intubation and transitioning to non-invasive ventilator-assisted breathing was a challenging task. An electronic fiber bronchoscope detected the presence of laryngomalacia (namely the supraglottic kind) and stenosis in the left bronchus. The patient underwent treatment involving tracheal intubation with a ventilator for a duration of 9 days, followed by the application of a non-invasive ventilator for an additional 9 days. On the eighteenth day of her admission, she underwent oxygen inhalation therapy until her discharge on the twenty-sixth day post-birth. When drinking milk, the swallowing function is poor, and the gastroesophageal reflux is prone to vomiting. Feeding can only be done through a nasal gastric tube. Nasal feeding is still required at her discharge. The patient also presents with slow weight gain and metabolic alkalosis that is difficult to correct. On the 26th day after birth, the body weight had increased only 130 grams.

The standard karyotype analysis revealed a karyotype of 46 XX 9qh+. Throughout her hospitalization, she underwent additional examinations. The postnatal brain color Doppler ultrasound revealed the absence of the septum pellucidum and dysplasia of the corpus callosum. Adrenal ultrasound showed that the left adrenal gland was larger than the right side. As a full-term child, two cardiac ultrasound examinations after birth revealed patent ductus arteriosus (4.5–5 mm). Automatic auditory brainstem response (AABR) showed that binaural hearing did not pass. There was no obvious abnormality in fundus screening.

**Figure 2:**
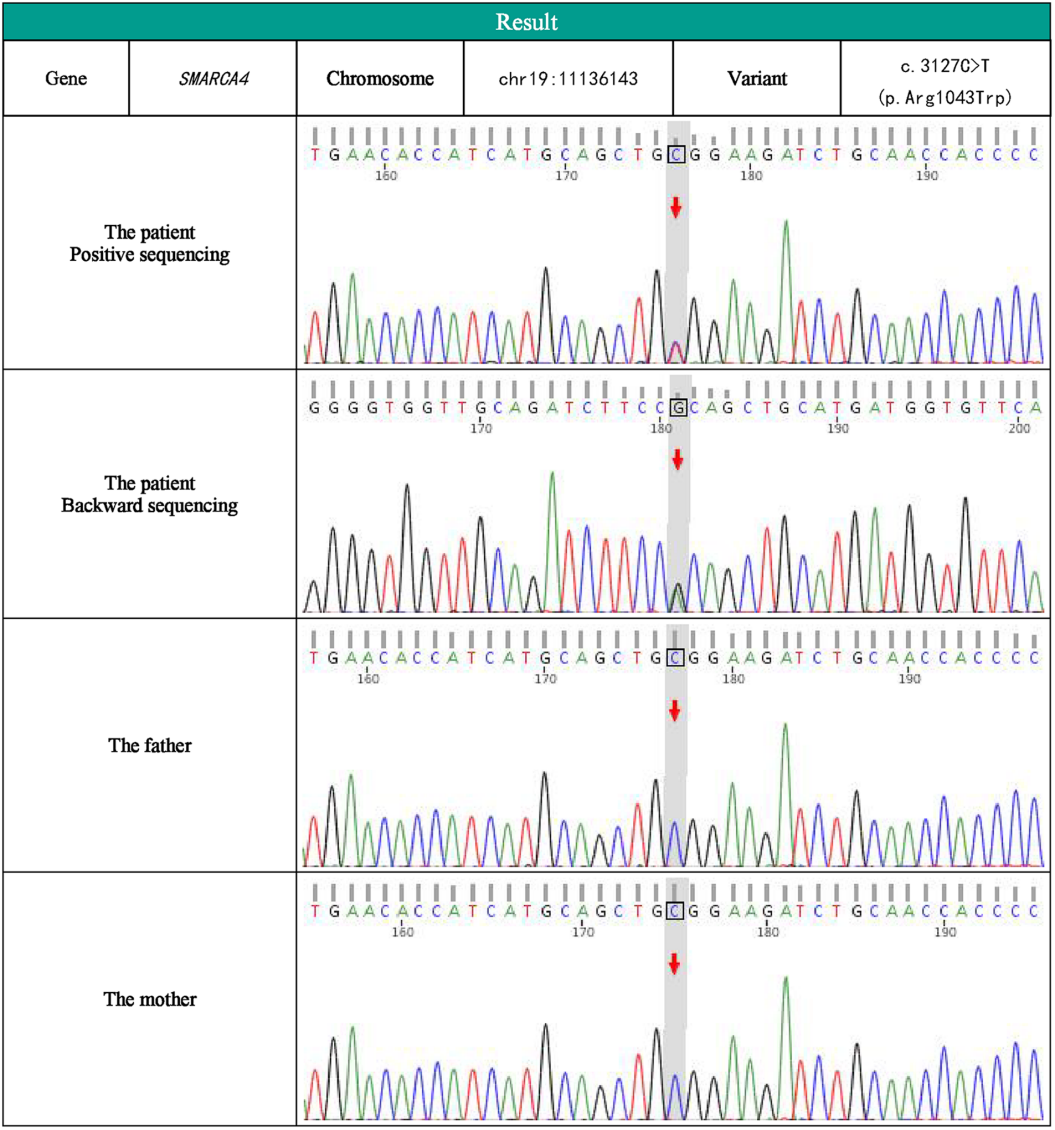
SMARCA2 SMARCA4 gene sequencing map of children and her parents.

We were interested in her anomalies (facial features, finger features, otolaryngologic features). Due to her cleft palate, upper airway obstruction, hypotonia, and finger deformity, we consider that there may be a genetic disease. To gain a better understanding of the disease's origin, a total of 2 ml of venous blood was collected from the youngster and his parents for whole exome sequencing at KingMed Diagnostics in China. The results showed that the patient had a heterozygous missense pathogenic variant in *SMARCA4* gene (NM_003072.5 c.3127C > T, p.Arg1043Trp). Sanger sequencing was used to verify the target sequence of the child and his parents. The verification results showed that there was a heterozygous pathogenic variant in the *SMARCA4* gene of the child with this problem, and the parents did not carry the pathogenic variant. It is part of a novel variant (Figure 2). The infant was diagnosed as as having CSS, *SMARCA4* pathogenic variant using exome sequencing.

This report indicates that, in accordance with the 2015 ACMG guidelines, the variant has been classified as “likely pathogenic” (PS2, PM2, PP3) based on the pathogenicity prediction provided by CADD (score = 47), and it has been recognized as “disease causing” by Mutation Taster.

### 2.2 Literature review

The common otorhinolaryngologic features of CSS include palate abnormalities, feeding difficulties, structural aerodigestive abnormalities, ear abnormalities, recurrent otitis media, and hearing loss. In the process of diagnosing this patient with CSS, we encountered documented cases in the literature that highlight the otolaryngologic manifestations associated with Coffin-Siris syndrome. Our case was genetically related to the *SMARCA4* gene. With “Coffin-Siris syndrome” and “*SMARCA4*” as the key words, the literature included in the PubMed database from the establishment of the database to July 2024 were searched (11–22). After excluding incomplete case reports and reading abstracts, we found 22 documented individuals with CSS in *SMARCA4* gene variant (11–15). We gathered 23 individuals, including this case (Table 1). A few patients with Coffin-Siris syndrome in *SMARCA4* gene variant had the same variation c.DNA, including c.2681C > T, c.2653C > T, c.2654G > A (Table 1). Our case variation c.DNA was c.3127C > T. She was different from the reported case gene variants. All these variations were heterozygous.

**Table 1:** Otolaryngologic, facial and finger features in patients with Coffin-Siris syndrome in *SMARCA4* gene variant.

Study	Facial features						Finger features		Otolaryngologic features						Developmental delay	Age	Sex	Variants c. DNA	Protein change
	Coarse appearance	Flat nasal bridge	Wide mouth	Thick lips	Thick eyebrows	Sparse scalp or thick hair	Digital anomalies	Hypotonia	Palatal abnormalities	Feeding difficulties	Ear abnormalities	Recurrent otitis media	Hearing loss	laryngotracheomalacia					
Tsurusaki etal. (11)	+	−	−	−	+	−	+	+	SMCP	unkown	−	+	−	−	+	18y	M	c.1636_1638del	p.Lys546del
Tsurusaki et al. (11)	−	−	−	+	+	+	+	+	H	+	+	−	+	+	+	20y	M	c.2576C > T	p.Thr859Met
Tsurusaki et al. (11)	+	−	+	+	+	−	+	+	C	+	+	−	+	−	+	9y	M	c.2653C > T	p.Arg885Cys
Tsurusaki et al. (11)	+	−	−	+	+	+	+	−	H	+	+	−	−	+	+	11y	M	c.2761C > T	p.Leu921Phe
Tsurusaki et al. (11)	−	+	+	+	+	−	+	+	H	+	+	−	+	−	+	16y	F	c.3032T > C	p.Met1011Thr
Tsurusaki et al. (11)	−	+	−	+	+	+	+	−	C	+	+	−	−	+	+	4y	M	c.3469C > G	p.Arg1157Gly
Tsurusaki et al. (15)	+	+	+	+	+	+	+	+	H	+	+	−	+	−	+	3y	M	c.2654G > A	p.Arg885His
Tsurusaki et al. (15)	+	+	+	+	+	−	+	−	C	+	+	−	−	−	+	6m	F	c.1372_1395del	p.Lys458_Glu465del
Tzeng et al. (14)	+	+	+	−	−	−	+	−	−	−	+	−	+	−	+	7y	M	c.2434C > T	p.Leu812Phe
Li et al. (12)	−	−	−	−	−	−	+	−	H	+	−	−	+	−	+	5y	F	c.3728G > A	p.Arg1243Glu
Li et al. (12)	+	−	−	−	+	+	+	+	C	unkown	−	−	+	−	+	8y	F	c.3512T > G	p.Val1171Gly
Li et al. (12)	+	+	−	+	−	−	+	−	unkown	−	+	−	unkown	−	+	8.5m	F	c.2653C > T	p.Arg885CysIle
Li et al. (12)	−	−	−	−	+	−	not noted	−	−	−	+	−	−	−	+	1.5y	M	c.3641T > C	p.Ile1214Thr
Li et al. (12)	−	−	+	−	−	+	+	−	−	−	+	−	+	−	+	2.5y	F	c.2654G > A	p.Arg885His
Li et al. (12)	−	−	+	−	−	+	+	+	−	−	+	−	−	−	+	3y	F	c.2681C > T	p.Thr894Met
Li et al. (12)	−	−	+	−	+	−	not noted	+	unknown	+	−	−	+	−	+	5y	F	c.1351C > T	p.Arg451Cys
Li et al. (12)	−	−	−	−	−	−	not noted	+	C	+	−	−	+	−	+	33m	M	c.2936G > A	p.Arg979Glu
Li et al. (12)	−	−	−	−	−	−	not noted	+	C	+	+	−	+	−	+	15m	F	c.2681C > T	p.Thr894Met
Li et al. (12)	−	−	−	−	−	−	not noted	−	unknown	+	+	−	+	−	+	2y	F	c.2851G > A	p.Gly951Arg
Li et al. (12)	+	−	−	−	−	−	+	−	SMC	+	+	−	+	−	+	16y	M	c.2900G > C	p.Gly883Ser
Li et al. (12)	−	−	−	−	−	+	not noted	−	H	+	−	−	−	−	+	16y	M	c.3508A > G	p.Thr1170Ala
Nikita et al. (13)	+	−	+	+	+	+	+	−	−	−	+	−	+	−	+	3y	M	c.2647G > A	p.Gly883Ser
Our case	+	+	+	+	+	+	+	+	C	+	Not clear	−	+	+	follow-up	New	F	c.3127C > T	p.Arg1043Trp

+, present; −, absent; C, cleft palate; H, high palate; SMCP, submucosal cleft palate.

We compared our case with the previous report with the same variant. The otolaryngologic, facial and finger features of CSS with the *SMARCE4* gene were shown in Table 1. In this review, the proportion of male patients was higher. Only our case was newborn, and other reported patients were children. Palatal abnormalities in these CSS patients with the *SMARCE4* gene variant mainly included cleft palate, high palate and submucosal cleft palate, of which the first two abnormalities were more common. We speculated that palatal abnormalities may be important clinical manifestations of CSS patients in *SMARCA4* gene variant. In addition to palatal abnormalities, they also seemed to be prone to have ear abnormalities, feeding difficulties, and hearing loss. Feeding difficulties were common in patients with palatal abnormalities. Laryngeal softening can occur in CSS patients. These two symptoms may persist until they grow up, not only newbron period. Ear abnormalities and hearing loss were also common otolaryngologic features of CSS. In these patients, some showed external ear abnormalities, while some of them displayed internal auditory canal stenosis. A few patients had recurrent otitis media. Our patients had no external ear malformations, unfortunately, we did not do further examination to assess the presence of internal auditory canal stenosis and other ear malformations. These otolaryngological abnormalities accounted for a high proportion in clinical symptoms.

All the 23 patients had different degrees of developmental delay. Our patient was a newborn who also had coarse appearance, digital anomalies, and developmental delay after birth. It is probable that she will experience intellectual disability in the future. She is currently in the follow-up stage. Most patients had digital anomalies, except those with unnoticed fingers. The common facial features of 23 cases with CSS in *SMARCA4* gene variant included coarse appearance, thick eyebrows. The typical facial features of CSS did not seem to be so obvious. Nearly half of them had hypotonia.

## 3 Discussion

Most CSS instances are primarily caused by new pathogenic variants, which are mostly sporadic in nature. The clinical manifestations of this condition are varied, characterized by distinct facial traits and abnormalities affecting multiple systems. It has very important value for diagnosis. Before the molecular basis is unclear, the diagnosis of CSS is based on clinical diagnostic criteria. Due to the rise of extensive genetic testing methods like whole exome sequencing, there is an increasing number of individuals with pathogenic variants in this pathway, leading to the expansion of the range of characteristics associated with CSS. Whole exome sequencing might be an efficient methodology to pinpoint the causal pathogenic variants.

This case had many common features (coarse appearance, hypotonia, finger deformity, hirsutism, corpus callosum dysplasia) with all previously reported CSS cases, and showed otolaryngologic abnormalities, including cleft palate, laryngomalacia, bronchial stenosis, feeding difficulties. The CSS patient is currently under follow-up. With the increase of age, the subsequent growth and development issues may show the evolution of CSS features.

The cleft palate associated with Coffin Siris syndrome has been described for a long time by some authors. In 2020, a study by Leiden Reed et al. presented a case of Coffin-Siris syndrome in a 5-year-old boy. The researchers highlighted the abnormal palatal process of *SMARCE1* pathogenic variant as a prominent characteristic of this genotype (23). Compared with the overall CSS patients, the reported *SMARCE1*-related CSS patients had a higher incidence of otolaryngologic features including palate abnormalities, feeding difficulties, and ear malformations.

The case we reported had the characteristic facial abnormalities and finger deformities of CSS, and the prominent otolaryngologic feature was cleft palate. This case also had laryngotracheomalacia, feeding difficulties and hearing loss. She had more otolaryngological malformations. The characteristic of our case was that Coffin-Siris syndrome was diagnosed in the neonatal period, and the diagnosis was confirmed by whole exome sequencing soon after birth due to multiple malformations. The genetic variation of our CSS patient was associated with *SMARCA4*. Specifically, the loss of *SMARCA4* function was associated with alterations in signaling pathways that are vital for proper tissue development, including those governing epithelial-mesenchymal transition (24). These findings support the hypothesis that the pathogenic mutation of *SMARCA4* disrupts the molecular signals required for normal palate development, leading to the observed defects. This case had many common features with the previously reported *SMARCE4* pathogenic variant cases, which further characterized the performance of the pathogenic variant, indicating that palate abnormalities may be a significant feature of the genotype.

CSS is associated with the *de novo* impairment of *ARID1A, ARID1B, SMARCA2, SMARCA4, SMARCB1, SMARCE1, SOX11*, and *PHF6* (24). Based on a study of 172 cases documented in the literature, it has been found that *SMARCA4* is responsible for causing pathogenic damage in 7% of cases with CSS (24). *SMARCA4* is an epigenetic regulator and chromatin remodeling factor of the SWI/SNF protein complex family. It is believed that the dominant negative effect is responsible for the destruction of *SMARCA4* in CSS patients (24). Patients exhibiting CSS mutations associated with *SMARCA4* appear to demonstrate a heightened susceptibility to behavioral issues, may present with reduced roughness in their facial characteristics, and continue to experience hypoplasia of the fifth finger and nails (13, 24, 25). *SMARCA4*-related CSS is a pleiotropic disease, and its pathological and clinical features are constantly evolving. A few reported individuals do not show a clear genotype-phenotype correlation.

The patients with *SMARCA4* gene variants may not have the obvious typical appearance characteristics as other types of CSS (12, 17). The clinical appearance characteristics of CSS caused by *SMARCA4* gene may be milder. Previous analysis of genotype-phenotype correlation in CSS patients, *SMARCA4* variants had been shown to be associated with organ-related dysfunction, intellectual disability, developmental delay, and cancer (12, 17). More precisely, the individuals with reported *SMARCA4* pathogenic variants may show inconsistent cardiac malformations, behavioral and cognitive issues, skeletal abnormalities, and abdominal wall malformations. These disease characteristics may be a result of random movements in gene expression that surpass the disease threshold (26). This has been observed in model organisms with trait penetrance (27). In this literature review, we found that the otolaryngologic features may also be the main clinical manifestations of CSS in *SMARCA4* gene variant.

We found a CSS neonatal case with *SMARCA4* pathogenic variation, which enriched the phenotype spectrum of *SMARCA4*-related CSS. As clinical genome sequencing is increasingly used to diagnose rare diseases such as CSS, we need new ways to explain the variation. Early improvement of gene sequencing is crucial for definitively diagnosing congenital otolaryngologic disorders in babies, especially when CSS is suspected.

This study presents a number of limitations. The limited sample size constrained the generalizability of our findings to the broader population of patients with CSS. Furthermore, long-term follow-up data were absent, and it was possible that some previous case reports had not been included in the literature review, which affected the generalization and reliability of our research findings.

The outcome of CSS is determined by the extent of organ involvement. At present, there exists no effective treatment option for individuals diagnosed with CSS. The approach to managing patients diagnosed with CSS involves symptomatic treatment, including rehabilitation therapy, growth hormone therapy, and the prevention of complications affecting the heart, gastrointestinal, and neurological systems (3, 28).

## 4 Conclusion

Coffin-Siris syndrome is a rare genetic disease inherited in an autosomal-dominated manner. It is often associated with malformations in the otorhinolaryngologic system. This case has many common features with previously reported CSS cases with pathogenic variant in the *SMARCA4* gene, which further characterizes the performance of the pathogenic variant, suggesting that palatal abnormalities may be a significant feature of the genotype. For patients with developmental abnormalities, whole-genome sequencing or whole-exome sequencing is particularly important to assist diagnosis. Currently, there is no known treatment for CSS, and individuals with CSS experience various complications affecting multiple systems. Due to the higher rate of related otorhinolaryngologic features, CSS may require more otolaryngology-related patient care.

## Data Availability

The datasets presented in this study can be found in online repositories. The names of the repository/repositories and accession number(s) can be found in the article/Supplementary Material.
